# Peripheral edema: A common and persistent health problem for older Americans

**DOI:** 10.1371/journal.pone.0260742

**Published:** 2021-12-16

**Authors:** Soroush Besharat, Hanna Grol-Prokopczyk, Shan Gao, Changyong Feng, Frank Akwaa, Jennifer S. Gewandter

**Affiliations:** 1 Department of Anesthesiology and Perioperative Medicine, University of Rochester, Rochester, NY, United States of America; 2 Department of Sociology, University at Buffalo, SUNY, Buffalo, NY, United States of America; 3 Department of Biostatistics and Computational Biology, University of Rochester, Rochester, NY, Rochester, NY, United States of America; 4 Division of Hematology and Oncology, Department of Medicine, University of Rochester, Rochester, NY, United States of America; Cardiff University, UNITED KINGDOM

## Abstract

Peripheral edema (i.e., lower limb swelling) can cause pain, weakness, and limited range of motion. However, few studies have examined its prevalence in the U.S. or its association with demographics, comorbidities, activity, or mobility. This study used data from the Health and Retirement Study, a nationally representative longitudinal survey of U.S. adults (age 51+/ N = 19,988 for 2016), to evaluate time trends and correlates of peripheral edema using weighted descriptive statistics and logistic regressions, respectively. Peripheral edema was assessed with the question “Have you had… // Persistent swelling in your feet or ankles?” The weighted prevalence of edema among older U.S. adults was 19% to 20% between 2000 and 2016. Peripheral edema was associated with older age, female sex, non-white race, low wealth, obesity, diabetes, hypertension, pain, low activity levels, and mobility limitations (odds ratios ranging from 1.2–5.6; p-values ≤0.001). This study provides the first estimates of national prevalence and correlates of peripheral edema among older Americans. Peripheral edema is common and strongly associated with comorbidities, pain, low activity levels, and mobility limitations, and disproportionately affects poorer and minority groups. Peripheral edema should be a focus of future research in order to develop novel and cost-effective interventions.

## Introduction

Chronic edema of the lower limbs (i.e., chronic peripheral edema) can cause pain, heaviness, weakness, discomfort, and negative body image, and limitations in mobility and flexibility [1]. Multiple causes of peripheral edema exist, including deficiencies in the venous or lymphatic systems [2], heart failure [3], and certain medications [4]. When peripheral edema is untreated, it can increase the risk of infection and ulcers [5]. Research performed in clinic-based samples or in countries outside the U.S. suggests that edema affects many individuals, including those with obesity, older age, sedentary lifestyles, history of deep venous thrombosis, and occupations that require long hours of standing [6, 7]. No cure for peripheral edema exists. Some forms of edema are responsive to diuretics [7], but these medications cause frequent urination that can lead to dehydration and decreased renal function. Dietary restrictions, such as limiting salt intake, can reduce edema as well. However, lifestyle changes are challenging to implement and sustain, especially for lower income individuals for whom easy access to healthful foods may be limited. Thus, chronic peripheral edema is likely left unaddressed in many individuals. Considering the high rates of obesity and other risk factors in the U.S., peripheral edema is likely a large public health challenge [7], but its overall prevalence among older adults in the U.S. is largely unknown.

The goals of this study were to use data from a nationally representative survey to (a) characterize the prevalence of chronic lower limb edema and recent time trends among older Americans, and (b) identify associations between peripheral edema and demographic and clinical features, activity levels, and mobility limitations in the U.S.

## Methods

### Study design

This study is a secondary analysis of the Health and Retirement Study (HRS) [8]. The HRS is sponsored by the National Institute on Aging (NIA U01AG009740) and conducted by the University of Michigan. HRS respondents are interviewed by telephone or in-person every two years and followed through multiple survey waves until their non-response or death. The HRS began in 1992, but was expanded to be nationally representative of the age 51+ non-institutionalized U.S. population in 1998. The sample is refreshed every 6 years to remain representative of this age range (although in interim years individuals under 55 are underrepresented). We used data from 2000, 2004, 2008, 2012, and 2016 to characterize time trends in the prevalence of peripheral edema among individuals age 55+; and data from the 2016 wave to investigate demographic and clinical factors associated with peripheral edema among individuals age 51+. All data were fully anonymized and the University of Rochester IRB declared the study exempt and waived the requirement for informed consent.

### Measures

The primary outcome was peripheral edema, assessed with the question, “Have you had any of the following persistent or troublesome problems: Persistent swelling in your feet or ankles?” (referred to as peripheral edema herein). If the respondent had previously completed the HRS survey, “since we last talked to you” was added to the end of the question stem. The use of the word “persistent” reduces the likelihood that respondents reported sporadic swelling events, increasing the validity of the question as an indicator of chronic peripheral edema. This question was fielded every 4 years between 2000 and 2016.

Demographic variables included in the analyses were age, sex, race, and wealth. Wealth rather than income was included as a measure of socioeconomic status based on evidence that the relationship between income and socioeconomic status is not linear in populations of mixed retirement status like those represented in the HRS [9]. The total household wealth variable was obtained from the RAND HRS Longitudinal File [10]. The variable “Total Wealth” is constructed by RAND by calculating the sum of all wealth components (including primary and secondary residences and other real estate, vehicles, businesses, investments, and other savings) minus all debt (including mortgages, other home loans, and all other debt). A detailed description of the imputation process for wealth measures is provided by RAND authors [11]. For purposes of these analyses, the wealth variable was separated into quartiles.

The self-reported clinical variables included BMI (calculated from self-reported height and weight), history of diabetes, history of hypertension, and pain. For questions related to diabetes and hypertension, the survey responses, “Disputes previous wave record, but now has condition” and “Disputes previous wave record, does not have condition” for the diabetes and hypertension variables were sorted into the “yes” and “no” groups, respectively. The HRS asks two questions related to pain: “Are you often troubled with pain?” and if yes, then “How bad is the pain most of the time: mild, moderate, or severe?” These two pain questions were merged into a single pain variable with four responses: no pain, mild pain, moderate pain, and severe pain [12]. These questions refer to pain in general and not necessarily pain associated with the lower limbs or edema.

Physical activity levels were estimated using the common question stem “How often do you take part in sports or activities that are…”, followed up by “…vigorous, such as running or jogging, swimming, cycling, aerobics or gym workout, tennis, or digging with a spade or shovel?”, “…moderately energetic such as, gardening, cleaning the car, walking at a moderate pace, dancing, floor or stretching exercises?”, and “…mildly energetic, such as vacuuming, laundry, home repairs?”. Responses to the physical activity questions included: more than once a week, once a week, one to three times a month, and hardly ever or never. Participants volunteering a response of “every day” were included in the “more than once a week” category.

Mobility limitations were estimated using the common question stem “Because of a health problem, do you have any difficulty with…?”, followed by “…walking several blocks,” “climbing several flights of stairs without resting,” and “getting up from a chair after sitting for long periods.” Participants volunteering the responses “can’t do” or “don’t do” were included in the yes category. For most variables (swelling, diabetes, hypertension, pain, mobility limitations, and physical activity), a small number of respondents (282–723 or 1.4%-3.6%) were excluded from analysis due to responses of “don’t know”, “not ascertained”, or “refused”.

### Analyses

The prevalences of peripheral edema, demographic and clinical features, activity, and mobility limitations were estimated using sampling weights. Sampling weights were necessary to obtain results that are representative of the older adult US population because the HRS oversamples groups including African Americans, Hispanics, and households with extremely frail respondents.(10) The comparison of prevalences over time (2000–2016) was restricted to respondents 55 years and older, since this age range is represented in every survey wave [13]. Cross-sectional analyses of the 2016 wave (the most recent wave with edema information) were used to investigate relationships between peripheral edema and other variables; these analyses included all available respondents (ages 51+).

Sample sizes for respondents aged 55 and above with weight coefficients available ranged from 17,061 to 18,837 between 2000 and 2016, representing population sizes of 56,102,332 to 93,562,417 older Americans in different years. Data were available from greater than 99.7% of respondents for the prevalence calculations for all years. The 2016 wave of the survey, used for all cross-sectional analyses, had 19,988 total respondents (age 51+), representing a population of 109,646,587.

Univariate statistics were used to compare the demographic, clinical, activity level, and mobility limitation variables between respondents who did or did not report persistent swelling by chi-square test (for categorical variables) or two-sample t-test (for continuous variables). A weighted multivariate logistic regression model was used to evaluate associations between demographic, clinical, activity level, and mobility limitation variables and peripheral edema (dependent variable), with missing data excluded via listwise deletion. A single multivariate logistic regression model was used to investigate the associations between all 3 activity level variables and peripheral edema (dependent variable), with adjustment for demographic and clinical variables. Separate weighted multivariate logistic regression models were used to assess the association between peripheral edema and each physical limitation variable (dependent variables), with adjustment for demographic and clinical variables in all models. The variance inflation factors for all variables used in the multivariate models were under 2, indicating that the multicollinearity among covariates is not problematic. The number of subjects used thus varied slightly for each analysis depending on the amount of missing data. The significance level was set at 0.05. No adjustments were made for multiplicity. All analyses were run using Stata/MP 15.1.

## Results

The prevalence of lower limb edema among older U.S. adults was 20.0%, 19.4%, 19.0%, 19.0%, and 19.1%, in 2000, 2004, 2008, 2102, and 2016, respectively. The prevalence of lower limb edema was strongly and significantly associated with all tested demographic, clinical, activity, and mobility limitation variables. Specifically, edema was associated with older age, female sex, minority race, low wealth, overweight/obese status, diabetes, hypertension, pain, low activity, and mobility limitations (Table 1 and S1 Table). These significant associations with demographic and clinical variables persisted in a multivariable logistic model including all variables simultaneously (Table 2). In particular, respondents over 90 years old and those 80–89 years old had 5.0 and 2.9 times the odds, respectively, of reporting peripheral edema compared to those who were 51–69 years old. Those in the wealthiest quartile were least likely to report peripheral edema (OR: 0.51 (95%CI: 0.43–0.61) 4^th^ vs. 1^st^ quartile). Obese individuals had 2.3 times the odds of reporting peripheral edema compared to those in the normal weight range. Finally, pain severity was particularly predictive of peripheral edema in the multivariable models. Compared to respondents with no pain, those with mild, moderate, or severe pain had 2.1, 2.85, and 5.61 times the odds of swelling, respectively (Table 2).

**Table 1:** Demographic and clinical characteristics (age 51+, 2016 HRS wave).

	Persistent Swelling in Feet or Ankles N = 4,456			No Persistent Swelling in Feet or Ankles N = 15,532			p-value
	Proportion or mean (SD), sample weight adjusted	Proportion or mean (SD), unadjusted	N	Proportion or mean (SD), sample weight adjusted	Proportion or mean (SD), unadjusted	N	
Age (years)	67.6 (12.8)	68.3 (11.9)	4456	64.5 (9.6)	65.7 (10.7)	15532	<0.0001
Age (decades)							
51–59	0.32	0.30	1,353	0.38	0.36	5,537	<0.0001
60–69	0.29	0.25	1,136	0.35	0.31	4,760	
70–79	0.22	0.23	1,025	0.18	0.21	3,187	
80–89	0.14	0.18	783	0.08	0.11	1,786	
90+	0.04	0.04	159	0.02	0.02	262	
Sex							
Male	0.39	0.33	1,478	0.49	0.45	6,957	<0.0001
Female	0.61	0.67	2,978	0.51	0.55	8,575	
Race							
White	0.73	0.61	2,701	0.81	0.68	10,548	<0.0001
Black / AA	0.17	0.28	1,245	0.10	0.20	3,097	
Other	0.11	0.11	493	0.09	0.12	1,829	
Ethnicity							
Non- Hispanic	0.89	0.83	3,718	0.90	0.84	13,073	0.2411
Hispanic	0.11	0.17	738	0.10	0.16	2,459	
Wealth (Quartiles)*							
1^st^ (lowest)	0.38	0.43	1,886	0.22	0.28	4,297	<0.0001
2nd	0.27	0.28	1,238	0.24	0.27	4,096	
3rd	0.20	0.18	801	0.26	0.24	3,648	
4^th^ (highest)	0.14	0.11	502	0.27	0.22	3,307	
BMI	31.5 (8.4)	31.4 (7.5)	4,371	28.2 (5.5)	28.3 (5.7)	15,295	<0.0001
BMI (Categorized)							
Underweight	0.02	0.02	68	0.01	0.02	252	<0.0001
Normal	0.17	0.17	750	0.27	0.27	4,125	
Overweight	0.29	0.29	1,282	0.39	0.38	5,888	
Obese	0.53	0.52	2,271	0.32	0.33	5,030	
History of diabetes							
No	0.66	0.63	2,807	0.80	0.77	11,892	<0.0001
Yes	0.34	0.37	1,643	0.20	0.23	3,623	
History of hypertension							
No	0.28	0.24	1054	0.49	0.44	6843	<0.0001
Yes	0.72	0.76	3394	0.51	0.56	8658	
Pain							
No pain	0.36	0.35	1564	0.66	0.65	10104	<0.0001
Mild Pain	0.14	0.14	613	0.11	0.11	1674	
Moderate Pain	0.33	0.32	1415	0.18	0.19	2874	
Severe Pain	0.17	0.19	816	0.04	0.05	804	

*The quartiles were created using the weighted data. AA: African American.

**Table 2:** Results of a weighted multivariable logistic regression of peripheral edema on demographic and clinical variables (age 51+, 2016 HRS wave).

Outcome: Persistent swelling in feet or ankles (N = 19,441)			
	Odds ratio	95% Confidence Limits	P-value
Age			
60–69 (vs. 51–59)	1.01	0.88, 1.16	0.8911
70–79 (vs. 51–59)	1.61	1.37, 1.88	<0.0001
80–89 (vs. 51–59)	2.91	2.45, 3.46	<0.0001
90+ (vs. 51–59)	5.03	3.91, 6.48	<0.0001
Sex			
Male (vs. Female)	0.78	0.69, 0.88	0.0001
Race			
Black / African American (vs. white)	1.42	1.25, 1.61	<0.0001
Other (vs. white)	1.24	1.03, 1.50	0.0250
Ethnicity			
Hispanic (vs. Non-Hispanic)	0.92	0.80, 1.05	0.2075
Wealth (Quartiles)			
2nd Q (vs. 1^st^ Q)	0.76	0.67, 0.86	0.0000
3^rd^ Q (vs. 1^st^ Q)	0.61	0.54, 0.70	0.0000
4^th^ Q (vs. 1^st^ Q)	0.51	0.42, 0.60	0.0000
BMI			
Underweight (vs. Normal)	1.11	0.73, 1.69	0.6261
Overweight (vs. Normal)	1.24	1.06, 1.47	0.0099
Obese (vs. Normal)	2.33	2.00, 2.70	<0.0001
History of diabetes			
Yes (vs. No)	1.22	1.09, 1.36	0.0009
History of hypertension			
Yes (vs. No)	1.5	1.30, 1.73	<0.0001
Pain (vs. No pain)			
Mild	2.1	1.79, 2.46	<0.0001
Moderate	2.84	2.49, 3.24	<0.0001
Severe	5.61	4.73, 6.65	<0.0001

*Results of single multivariable regression model including all variables simultaneously.

In a multivariable model adjusting for all demographic and clinical variables, less frequent exercise (whether mild, moderate, or vigorous) was significantly associated with peripheral edema (Table 3). For example, reporting mild, moderate, and vigorous exercise more than once a week resulted in considerable decrease in the odds of reporting peripheral edema when compared to never performing that level of exercise (OR mild: 0.67 (95%CI: 0.56–0.79); OR moderate: 0.76 (95%CI: 0.65–0.89); OR vigorous: 0.71 (95%CI: 0.61–0.83)). Similar models also identified strong associations between peripheral edema and difficulty standing up from a chair (OR: 2.20 (95% CI: 1.95–2.48)), difficulty climbing several flights of stairs (OR: 2.33 (95% CI: 2.06–2.64)), and difficulty walking several blocks (OR: 2.98 (95% CI: 2.60–3.40)) (Table 4).

**Table 3:** Results of a weighted multivariable logistic regression analysis of peripheral edema on activity level, adjusted for demographic and clinical variables (age 51+, 2016 HRS wave).

Outcome: Persistent swelling in feet or ankles(N = 19,281)			
	Odds ratio	95% Confidence Limits	P-value
**Vigorous exercise**			
1–3 times per month (vs. Never)	0.78	0.65, 0.95	0.0115
Once a week (vs. Never)	0.66	0.55, 0.79	<0.0001
> once a week (vs. Never)	0.71	0.61, 0.83	<0.0001
**Moderate exercise**			
1–3 times per month (vs. Never)	0.91	0.77, 1.06	0.2124
Once a week (vs. Never)	0.91	0.77, 1.07	0.2587
> once a week (vs. Never)	0.76	0.65, 0.89	0.0011
**Mild exercise**			
1–3 times per month (vs. Never)	0.71	0.54, 0.92	0.0117
Once a week (vs. Never)	0.75	0.63, 0.89	0.0012
> once a week (vs. Never)	0.67	0.56, 0.79	0.0000

* Result for a single multivariate model that adjusts for age, sex, race, ethnicity, BMI, history of diabetes, history of hypertension, pain, and wealth (quartiles).

**Table 4:** Results of weighted multivariable logistic regression models of mobility limitations, on peripheral edema adjusted for demographic and clinical variables (2016 HRS wave).

Outcome	Odds ratio	95% Confidence Limits	P-value
**Difficulty standing up from a chair** (N = 19,375)			
Persistent ankle or foot swelling (Yes vs. No)	2.2	1.95, 2.48	<0.0001
**Difficulty climbing several flights of stairs** (N = 19,370)			
Persistent ankle or foot swelling (Yes vs. No)	2.33	2.06, 2.65	<0.0001
**Difficulty walking a few blocks** (N = 19,293)			
Persistent ankle or foot swelling (Yes vs. No)	2.97	2.60, 3.40	<0.0001

* Result for each mobility limitation is from a separate multivariable model that adjusts for age, sex, race, ethnicity, BMI, history of diabetes, history of hypertension, pain, and wealth quartile.

## Discussion

To our knowledge, this is the first population-based study and first study conducted in the U.S. to investigate the prevalence of peripheral edema. Other studies have investigated the prevalence of chronic edema or lymphedema in hospital- and primary care clinic-based populations in Europe [14, 15]. However, potential selection bias from clinic-based recruitment limits the generalizability of these results to the general population. Our data are directly relevant to and representative of the U.S. older adult population (i.e., 55+ years for prevalence time trends) and identify a staggering prevalence of peripheral edema (i.e., 19%-20% depending on the year). As expected, this prevalence is lower than estimates obtained in the clinic-based studies (e.g., 38% [14] and 56% [15]), but still considerable. In addition to documenting the persistently high prevalence of lower-limb edema in the U.S. between 2000 and 2016, this study found edema to be strongly associated with age, obesity, pain, less frequent physical activity, and mobility limitations (standing, walking, and climbing stairs). These associations confirm and strengthen the findings of previous studies in clinic-based populations in Europe [1, 14, 16, 17]. Although the cross-sectional associations identified in this study cannot elucidate causality, the fact that peripheral edema is consistently associated with many markers of ill health in multivariable models suggests that peripheral edema is potentially a highly detrimental problem. Thus, it needs to be addressed by primary care clinicians and should be a focus of future interventional research programs.

Sixty-five percent of respondents with peripheral edema reported having at least mild pain most of the time. This percentage is only slightly lower than the 80% of patients with peripheral edema from Canadian outpatient wound clinics who reported having at least some pain or discomfort [17]. We would expect that pain in the general population with persistent swelling would be lower than pain in patients whose edema is sufficiently severe to seek care at a wound clinic, especially if some of the pain is in fact from the peripheral edema. Experiencing even mild pain was associated with 2.1 times the odds of reporting persistent swelling, while respondents with severe pain had, strikingly, 5.6 times the odds of reporting swelling. These data suggest that edema could cause significant pain, a potential causal relationship that warrants future study. We also observed a generally consistent decrease in the likelihood of reporting peripheral edema with increased frequency of activity, regardless of the activity intensity. Notably, even reporting of infrequent exercise was associated with lower odds of reporting peripheral edema. For example, respondents who reported mild exercise just 1–3 times per month had 33% lower odds of reporting swelling than those who hardly ever or never took part in mild exercise. Thus, these data suggest that even very modest amounts of exercise may be effective at decreasing swelling. However, they could also suggest that peripheral edema prevents many people from performing even infrequent, mild exercise since these data cannot determine the causal direction. Individuals who reported persistent swelling had almost 3 times the odds of reporting having difficulty walking several blocks. These data are consistent with those from a large study of individuals recruited from UK Community Nursing Services that demonstrated that patients with peripheral edema more commonly required walking aides than those without peripheral edema [15]. Those who reported difficulty climbing stairs or standing up from a chair were at slightly less increased risk of reporting swelling (with odds ratios of 2.2 and 2.3, respectively). Although these data cannot determine causality, one possible explanation for the results is that persistent swelling plays a particularly significant role in difficulty walking. If this is the case, outcomes related to ability to walk or amount of walking would be important outcomes for trials of peripheral edema interventions. Difficulty walking was reported the least frequently of the three mobility limitations (i.e., 25% of respondents vs. 35% and 42% for standing up from a chair and climbing stairs, respectively), suggesting that individuals with difficulty walking several blocks might have the highest level of overall physical impairment, and that high levels of physical impairment have the strongest association with peripheral edema.

**Fig 1:**
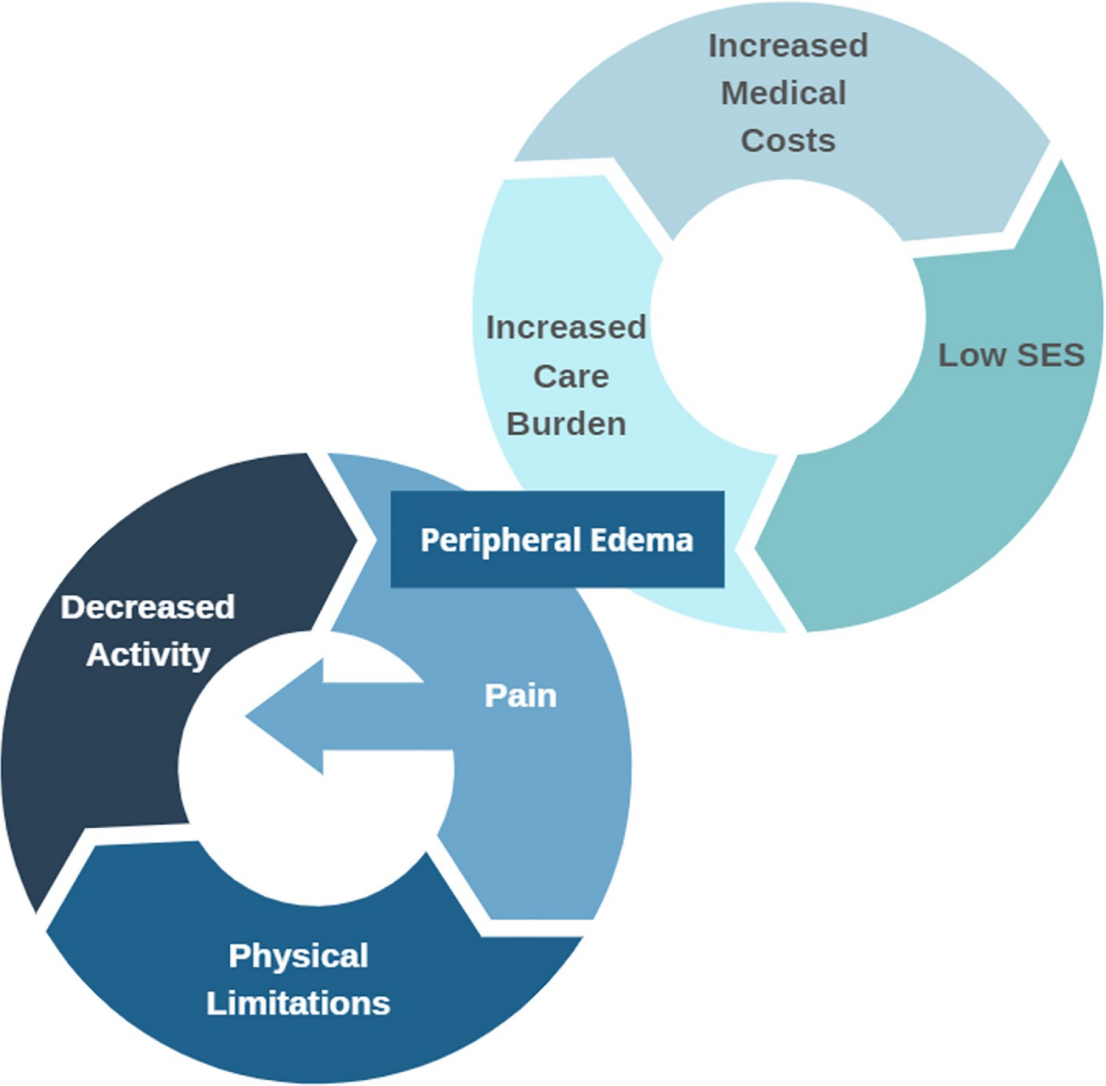
Conceptual model of vicious cycles of edema. This model hypothesizes that peripheral edema can cause pain, leading to physical limitations and decreased activity, which in turn would further increase edema. In addition, individuals with lower SES were more likely to have peripheral edema, which could lead to increased health care burden and medical costs, which can lead to further economic disadvantage.

The strong associations found in these data between peripheral edema and pain, low levels of physical activity, and physical limitations support a hypothetical “vicious cycle” model in which peripheral edema causes pain and limited physical activity, and in turn limited activity increases the severity of peripheral edema (Fig 1). Continuation of this vicious cycle could cause debilitating edema and impaired quality of life. The perpetuation of inactivity and mobility limitations can also contribute to comorbid conditions that were associated with peripheral edema in our study (i.e., obesity, diabetes, and hypertension). The prevalence of these conditions are, in fact, increasing in the U.S [18–20]. Future research that investigates the causal relationships between these domains are necessary to confirm the validity of this model.

Our findings also indicate that social disparities in lower limb edema are significant, with Blacks/African Americans and other racial minorities, women, and less wealthy individuals reporting the highest rates of peripheral edema. These patterns mirror those for high-impact chronic pain, disability, and obesity [21–24]. These disparities were observed in both bivariate associations (Table 1) and in a fully-adjusted model (Table 2), indicating that race, sex, and wealth predict risk of edema independently, and net of age, BMI, diabetes, hypertension, and pain level. Minority racial status and lower wealth could be associated with peripheral edema for multiple reasons, including higher rates of other chronic health conditions as well as lower access to healthy foods and preventive care services. The high prevalence of peripheral edema among disadvantaged groups is especially problematic because of the potential negative social and economic effects of chronic edema. These may include increased care burden on families, negatively affected relationships leading to social isolation, out-of-pocket treatment expenses, quality-of-life detriments, and loss of productivity [25]. These negative effects may contribute to another hypothetical “vicious cycle”, in which low socioeconomic status increases the likelihood of developing chronic edema which in turn leads to outcomes that negatively impact individual or family socioeconomic status (Fig 1). The causal nature of these relationships should be investigated in future studies. The sociodemographic disparities in peripheral edema found herein highlight the need for effective and affordable interventions for peripheral edema that are covered by insurance. Future research could clarify the precise mechanisms underlying social disparities in edema.

### Limitations

The primary limitation of this study is that the identified associations are cross-sectional, and thus cannot address causal relationships. Future research should investigate the causal direction of the relationships identified in this study. In addition, we did not investigate the distribution of potential causes of edema (e.g., heart failure [3] or medication usage [4]) in this cohort, which could provide important information regarding allocation of resources to treat edema. A single self-report question was used to identify peripheral edema. Although a clinician confirmed-diagnosis would have been ideal, the wording of the survey question (i.e., “*persistent* swelling of the feet and ankles”) likely prevented endorsement by respondents because of temporary swelling problems. The question did ask if the respondents had persistent swelling either with no time constraint or since they last spoke to the study team and so it is possible that respondents who had previous persistent swelling that had resolved would endorse swelling even if they currently did not have peripheral edema. However, we would expect that if this type of misclassification occurred, it would bias estimates of differences between groups toward the null, rather than increasing differences. The data are limited to older adults in the U.S. because of the nature of the data set. Future population-based research in other countries will be useful to characterize the global burden of peripheral edema. Finally, self-report of certain variables that we used are known to differ from objective measures, including physical activity levels [26] and BMI [27], so some measurement error is expected for these predictors.

## Conclusions

To our knowledge, this study is the first to provide national prevalence estimates of chronic peripheral edema among older U.S. adults. The results show that chronic peripheral edema is common and persistently affecting approximately 19% of the population. It also disproportionately affects women, racial minorities, and less wealthy individuals. People living with peripheral edema have more pain, comorbidities, and mobility limitations, and participate in less physical activity. In addition to lifestyle interventions, novel non-pharmacologic interventions would be ideal for the treatment of peripheral edema because of polypharmacy challenges that are common for older adults and individuals with multiple comorbid conditions. Peripheral edema should be a focus of clinicians and of future research in order to develop novel, non-pharmacologic and cost-effective interventions.

## Supporting information

S1 Table Demographic and clinical characteristics of all respondents (ages 51+, 2016 RHS wave). (DOCX)

S2 Table Activity and mobility limitations sample characteristics (age 51+, 2016 HRS wave). (DOCX)
