# Supplementary material for: Peripheral edema: A common and persistent health problem for older Americans
Source: PLoS One. 2021 Dec 16;16(12):e0260742. doi: 10.1371/journal.pone.0260742 (PMC8675752; doi:10.1371/journal.pone.0260742)
Supplement: S1 Table — (DOCX) [file pone.0260742.s001.docx]

Supplemental Table 1. Demographic and clinical characteristics of all respondents (Ages 51+, 2016 RHS wave)

|  | All Participants  N=19,988 | | |
| --- | --- | --- | --- |
|  | Proportion or mean (SD), sample weight adjusted | Proportion or mean (SD), unadjusted | N |
| Age (years) | 65.1 (10.3) | 66.3 (11.1) |  |
| Age (decades) | | | |
| 51-59 | 0.37 | 0.35 | 6,890 |
| 60-69 | 0.34 | 0.29 | 5,896 |
| 70-79 | 0.19 | 0.21 | 4,212 |
| 80-89 | 0.09 | 0.13 | 2,569 |
| 90+ | 0.02 | 0.02 | 421 |
| Sex | | | |
| Male | 0.47 | 0.42 | 8,435 |
| Female | 0.53 | 0.58 | 11,553 |
| Race | | | |
| White/  Caucasian | 0.80 | 0.67 | 13,249 |
| Black or African American | 0.11 | 0.22 | 4,342 |
| Other | 0.09 | 0.11 | 2,322 |
| Ethnicity | | | |
| Non-Hispanic | 0.90 | 0.84 | 16,791 |
| Hispanic | 0.10 | 0.16 | 3,197 |
| Wealth (Quartiles)* | | | |
| 1^st^ (lowest) | 0.25 | 0.31 | 6,183 |
| 2nd | 0.25 | 0.27 | 5,334 |
| 3rd | 0.25 | 0.22 | 4,449 |
| 4^th^ (highest) | 0.25 | 0.19 | 3,809 |
| BMI | 28.8 (6.2) | 28.9(6.3) | 19,666 |
| BMI (Categorized) | | | |
| Underweight | 0.01 | 0.02 | 320 |
| Normal | 0.25 | 0.25 | 4,875 |
| Overweight | 0.37 | 0.36 | 7,170 |
| Obese | 0.36 | 0.37 | 7,301 |
| History of diabetes | | | |
| No | 0.77 | 0.74 | 14,699 |
| Yes | 0.23 | 0.26 | 5,266 |
| History of hypertension | | | |
| No | 0.45 | 0.40 | 7,897 |
| Yes | 0.55 | 0.60 | 12,052 |
| Pain | | | |
| No pain | 0.61 | 0.59 | 11,668 |
| Mild Pain | 0.12 | 0.12 | 2,287 |
| Moderate Pain | 0.21 | 0.22 | 4,289 |
| Severe Pain | 0.07 | 0.08 | 1,620 |
