# Supplementary material for: Peripheral edema: A common and persistent health problem for older Americans
Source: PLoS One. 2021 Dec 16;16(12):e0260742. doi: 10.1371/journal.pone.0260742 (PMC8675752; doi:10.1371/journal.pone.0260742)
Supplement: S2 Table — (DOCX) [file pone.0260742.s002.docx]

Supplemental Table 2. Activity and mobility limitations sample characteristics (Age 51+, 2016 HRS wave).

|  | Persistent Swelling in Feet or Ankles  N=4,456 | | | No Persistent Swelling in Feet or Ankles  N=15,532 | | | p-value |
| --- | --- | --- | --- | --- | --- | --- | --- |
|  | Proportion or mean (SD), sample weight adjusted | Proportion or mean (SD), unadjusted | N | Proportion or mean (SD), sample weight adjusted | Proportion or mean (SD), unadjusted | N |  |
| Vigorous exercise (N=19,890) | | | | | | | |
| Hardly ever / never | 0.71 | 0.72 | 3,207 | 0.46 | 0.50 | 7,768 | <0.0001 |
| 1-3 times a month | 0.08 | 0.07 | 322 | 0.11 | 0.11 | 1,637 |  |
| Once a week | 0.07 | 0.07 | 296 | 0.12 | 0.12 | 1,837 |  |
| > once a week | 0.14 | 0.14 | 617 | 0.30 | 0.27 | 4,206 |  |
| Moderately energetic exercise (N=19,911) | | | | | | | |
| Hardly ever / never | 0.34 | 0.36 | 1,613 | 0.15 | 0.18 | 2,740 | <0.0001 |
| 1-3 times a month | 0.13 | 0.13 | 593 | 0.11 | 0.12 | 1,862 |  |
| Once a week | 0.17 | 0.17 | 732 | 0.17 | 0.18 | 2,768 |  |
| > once a week | 0.36 | 0.34 | 1,497 | 0.58 | 0.52 | 8,106 |  |
| Mildly energetic exercise (N=19,934) | | | | | | | |
| Hardly ever / never | 0.19 | 0.21 | 929 | 0.08 | 0.10 | 1,499 | <0.0001 |
| 1-3 times a month | 0.09 | 0.10 | 423 | 0.07 | 0.08 | 1,213 |  |
| Once a week | 0.27 | 0.28 | 1,262 | 0.23 | 0.26 | 3,960 |  |
| > once a week | 0.45 | 0.41 | 1,827 | 0.62 | 0.57 | 8,821 |  |
| Difficulty standing up from a chair (N=19,913) | | | | | | | |
| No | 0.36 | 0.34 | 1,521 | 0.72 | 0.69 | 10,693 | <0.0001 |
| Yes | 0.64 | 0.66 | 2,919 | 0.28 | 0.31 | 4,780 |  |
| Difficulty climbing several flights of stairs (N=19,902) | | | | | | | |
| No | 0.27 | 0.25 | 1,098 | 0.65 | 0.60 | 9,285 | <0.0001 |
| Yes | 0.73 | 0.75 | 3,341 | 0.35 | 0.40 | 6,178 |  |
| Difficulty walking a few blocks (N=19,826) | | | | | | | |
| No | 0.40 | 0.38 | 1,677 | 0.80 | 0.77 | 11,863 | <0.0001 |
| Yes | 0.60 | 0.62 | 2,732 | 0.20 | 0.23 | 3,554 |  |
